# Correlation of serum hepcidin levels with disease progression in hepatitis B virus-related disease assessed by nanopore film based assay

**DOI:** 10.1038/srep34252

**Published:** 2016-10-03

**Authors:** Jing Wang, Ailian Dong, Gang Liu, Gregory J. Anderson, Tony Y. Hu, Jian Shi, Yulin Hu, Guangjun Nie

**Affiliations:** 1 CAS Key Laboratory for Biomedical Effects of Nanomaterials & Nanosafety, Beijing Key Laboratory of Ambient Particles Health Effects and Prevention Techniques, CAS Center for Excellence in Nanoscience, National Center for Nanoscience and Technology, Beijing, China; 2 The First Affiliated Hospital of Jilin University, Changchun, Jilin Province, China; 3 Iron Metabolism Laboratory, QIMR Berghofer Medical Research Institute, Brisbane, Queensland, 4006 Australia; 4 Department of Nanomedicine, Houston Methodist Hospital Research Institute, Houston, TX, USA

## Abstract

Chronic hepatitis B virus (HBV) infection often develop into cirrhosis, and both are major risk factors of hepatocellular carcinoma. However, effective approaches for the monitoring of HBV-related disease progress are still in need. Increased iron storage has an important role in HBV-related diseases. Hepcidin is a key regulator of iron homeostasis whose expression changes are often indicative of abnormal iron metabolism. There are few reports of hepcidin levels in patients with HBV infections, and the available results are inconsistent. In this study, using a recently validated nanopore silica film based method, we measured serum hepcidin levels in 46 HBV-related patients and 20 healthy controls. Patients were divided into three groups: chronic hepatitis B without cirrhosis; HBV-related cirrhosis; and HBV-related cirrhosis with hepatocellular carcinoma. Compared to healthy controls, the mean serum hepcidin level was significantly higher in CHB patients without cirrhosis, and in those with hepatocellular carcinoma, but not in those with cirrhosis. Iron-loading, viral infection and liver dysfunction are determined to be the major regulators of hepcidin in these patients. These observations suggest correlations between serum hepcidin and progression of chronic HBV infection, and may shed a new light on the development of biomarkers for HBV-related disease surveillance.

It is estimated that there are approximately 450 million hepatitis B virus (HBV) carriers worldwide and 50 million new cases are added every year^1^,^2^, making chronic hepatitis B (CHB) one of the world’s most common diseases. Many patients with CHB develop liver cirrhosis, and this often progress to hepatocellular carcinoma (HCC)^3^. Both of these are associated with high morbidity and mortality. Currently chronic infection of HBV and subsequent cirrhosis is recognized as one of the most common risk factors of HCC^4^. Therefore, monitoring the disease progression of HBV infection is of great clinical importance both for the treatment of CHB and for the early diagnosis of related diseases. Unfortunately, development of non-invasive biomarkers for HBV infection progression has only achieved limited success. So far the only clinically available biomarker for HCC surveillance and diagnosis remains to be α-fetoprotein, which suffers from limited sensitivity and specificity^5^, and is particularly subject to interferences from other chronic liver diseases^4^. These suggested that further investigations of physiological and pathological changes during HBV infection is still needed for a better understanding of HBV-related diseases and for their better clinical surveillance and treatment.

Chronic viral hepatitis are often associated with iron overload^6^, which is also a major risk factor for HCC development. This may be related to the ability of iron to generate oxidative stress, leading to tissue damage and chronic inflammation in the liver^7^. Hepcidin, a 25-amino acid peptide primarily produced by hepatocytes^8^, is the central regulator of body iron homeostasis^9^. Hepcidin binds to the iron export protein ferroportin on the plasma membrane of a variety of cell types and facilitates its internalization and degradation, which subsequently reduces iron efflux from cells and leads to intracellular iron accumulation^10^. Therefore hepcidin is seen as a potential indicator of body iron status, and given the probable impact of HBV infection on patient iron metabolism, understanding hepcidin regulation in HBV-related diseases may provide important insight into the link between viral hepatitis, iron accumulation and HCC^11^.

Although methods for determining hepcidin levels in biological fluids, notably immunoassays and those based on mass spectrometry (MS), are becoming increasingly available^12^, accurate and facile hepcidin quantification remains a challenge. Many immunoassays, including early commercial ELISA kits, suffer from inaccurate results, which have been attributed to insufficient antibody specificity among N-terminal truncated isoforms of the peptide^13^. On the other hand, assays based on MS techniques, including matrix-assisted laser desorption/ionization time-of-flight (MALDI-TOF) MS, LC-MS/MS, or surface-enhanced laser desorption/ionization time-of-flight (SELDI-TOF) MS, despite being highly capable in discerning the isoforms, often require time-consuming sample pre-treatments.

In view of these difficulties, we have previously established a nanopore silica film based approach for hepcidin determination. The nanopores in the film captures and enriches hepcidin and other small peptides with appropriate size from physiological samples, and the captured peptides can be eluded and quantified by MALDI-TOF MS. We have demonstrated that this assay has a lower limit of quantification (<2 nmol/l in human serum) sufficient for clinical measurements and have successfully applied it to clinical samples^14^,^15^.

Up to now, investigations on the role of hepcidin in HBV-related diseases have been greatly hindered by the lack of appropriate hepcidin quantification method. Only a small number of previous studies have examined the relationship between hepcidin and chronic HBV infection. Given the challenges in quantification of the biologically active form of hepcidin, hepcidin-25, most of these studies are based on measurements of its precursor prohepcidin^16^,^17^,^18^,^19^. However, prohepcidin itself is not a functional iron regulator and its reliability as indicator of hepcidin-25 has been questioned^20^. Only two studies have measured biologically active serum hepcidin-25 in HBV infected patients, using immunoassays^21^,^22^, and their results still need further validation.

Herein, we applied our established nanopore film based assay to investigate serum hepcidin-25 levels in a cohort of patients with CHB, HBV-related cirrhosis or HBV-related cirrhosis with hepatocellular carcinoma (HBV-HCC), in order to better understand the associations between hepcidin and HBV-related diseases. This work may provide new insights into the role of iron metabolism in HBV infections, and into the development of new approaches for monitoring of HBV-related disease progression.

## Results

### Clinical profiles and levels of iron indicators of HBV-infected patients

The clinical features of the patients and controls involved in this study are summarized in Table 1. Subjects were well matched by gender and age. There were statistically significant differences between the groups with respect to HBV-DNA, alanine aminotransferase (ALT), albumin, hemoglobin, serum iron and ferritin levels. CHB patients showed a significantly higher HBV-DNA load than the other two patient groups, and significantly higher ALT levels than HCC patients. Non-cirrhotic CHB patients had higher albumin levels than patients with cirrhosis or HCC. CHB and HBV-HCC patients had significantly higher hemoglobin levels than those with cirrhosis. HBV-related patients had significantly higher serum iron and ferritin levels than healthy controls, and both the CHB group and the HCC group had significantly higher ferritin than the cirrhosis group. No significant differences in the inflammation marker CRP were found between the patient groups.

### Serum hepcidin levels at different stages of HBV infection

**Figure 1:**
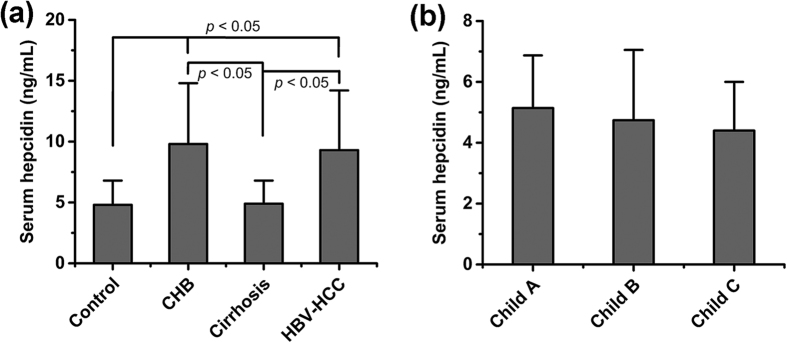
Hepcidin levels in HBV-infected patients. (**a)** Serum hepcidin concentrations in controls and patients with HBV-related disease. (**b**) Serum hepcidin in patients with HBV-related cirrhosis in relation to Child-Pugh Class. CHB, chronic hepatitis B; HBV-HCC, HBV-related cirrhosis with hepatocellular carcinoma.

Hepcidin measurements by the nanopore film based assay indicated significant intergroup differences in serum hepcidin levels (Fig. 1a). The mean hepcidin level was higher in both CHB (9.8 ± 5.0 ng/mL) and HBV-HCC patients (9.3 ± 4.9 ng/mL) than in healthy controls (4.8 ± 2.0 ng/mL). Interestingly, however, the mean serum hepcidin level of patients with HBV-related cirrhosis (4.9 ± 1.9 ng/mL) was not significantly different from controls. There were no statistically significant differences between patients in the various Child-Pugh groups (*p* > 0.05), but Child–Pugh class C patients had a slightly lower average value (Fig. 1b).

### Correlations between hepcidin and other clinical parameters

**Figure 2:**
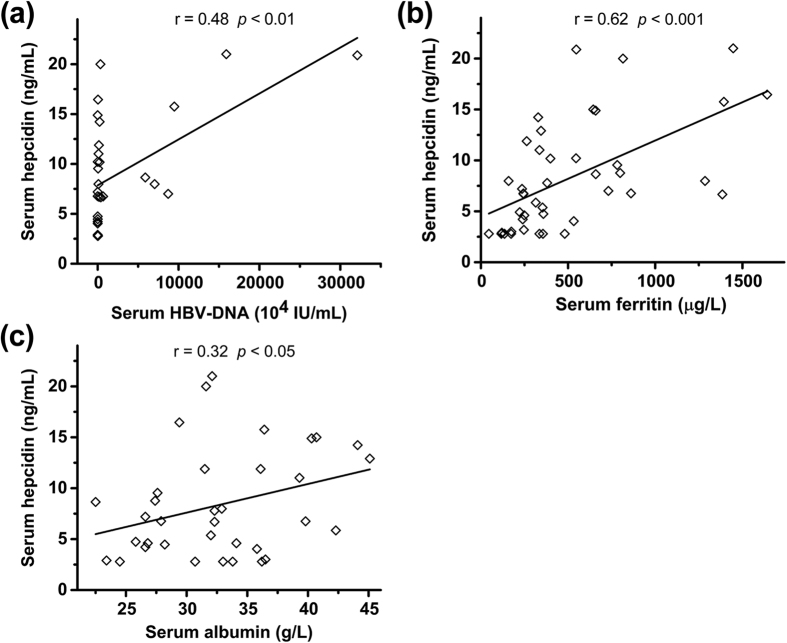
Correlations between hepcidin and clinical indices. (**a**) Between serum hepcidin and HBV-DNA load. (**b**) Between serum hepcidin and ferritin. (**c**) Between serum hepcidin and albumin.

Correlations between serum hepcidin levels and clinical indices are summarized in Table 2. Serum HBV-DNA (r = 0.48, *p* < 0.01; Fig. 2a), ferritin (r = 0.62, *p* < 0.001; Fig. 2b), and albumin (r = 0.32, *p* < 0.05; Fig. 2c) levels were positively correlated with hepcidin. Moreover, HBV-DNA loading was also positively correlated with hepcidin in the CHB and cirrhosis groups, and in the cirrhosis and HBV-HCC groups correlations between ferritin and hepcidin were observed. No correlation between serum hepcidin levels and other biochemical parameters, nor with age or gender in any group was observed. Multiple linear regression analysis (Table 3) confirmed that HBV-DNA, ferritin and albumin are independent predictors of hepcidin level (β = 0.702, *p* < 0.05; β = 0.516, *p* < 0.05; β = 0.363, *p* < 0.05, respectively).

## Discussion

There have been few previous studies on hepcidin expression in patients with chronic HBV infection, and only a very small number of these have measured biologically active hepcidin-25. Other studies have relied on measuring either liver hepcidin mRNA^23^, or the pre-peptide prohepcidin^16^,^17^,^18^,^24^. Hepcidin mRNA levels have been shown to be appropriate surrogates for hepcidin-25^25^, but to conduct these analyses requires invasive liver biopsy. Prohepcidin can be measured readily in serum or urine, but it is not particularly reliable in predicting hepcidin-25 levels^20^,^26^. Furthermore, some studies (e.g.^21^) that have measured hepcidin-25 employed an early commercial ELISA hepcidin kit, the ability of which to distinguish between iron metabolism disorders in some cases has been questioned^27^. The mass spectrometry based hepcidin assay we have developed overcomes these difficulties by directly measuring hepcidin-25.

Overall, our investigations demonstrated an increase in hepcidin expression in HBV-infected patients relative to controls, with the exception of patients in the cirrhosis group. These results are in accordance with Wang *et al*.^22^ who reported that serum hepcidin was upregulated in both CHB and HBV-HCC patients. Two studies have shown that serum prohepcidin is decreased in CHB^17^,^18^ relative to healthy controls, however, the physiological relevance of these results is questionable, as noted above. A single study has found hepcidin-25 to be down-regulated in patients with HBV-related cirrhosis relative to controls^21^, but in this study the focus was on cirrhosis per se, and patients with active HBV infection were excluded, so it is not directly comparable with our own.

The major factors that affect hepcidin levels are iron stores, inflammation, hypoxia and erythropoietic activity^28^. Correlations between iron parameters and hepcidin are often observed with chronic viral hepatitis. Aoki *et al*. reported a significant positive correlation between hepcidin mRNA expression and serum iron levels (but not inflammation) in CHC^29^, and similar associations have been reported with serum ferritin and/or liver iron stores^30^,^31^. However, the relationship between hepcidin and CHB is less clear. Nagashima *et al*. found that serum ferritin and prohepcidin levels were positively associated in CHB patients^16^, and Wang *et al*. reported positive correlations between serum hepcidin-25 and ferritin in CHB and HBV-HCC^22^. Other studies, using prohepcidin, found either no association with iron parameters in HBV^17^ or a negative correlation^18^.

Our results showed that non-cirrhotic CHB patients had increased hepcidin compared to controls. Many studies have shown that the production of hepcidin is stimulated by inflammation^8^,^32^. The pro-inflammatory cytokine IL-6 is a strong stimulator of hepcidin expression^33^, and in CHB patients IL-6 levels are higher than those of healthy controls^34^. In addition, viral or bacterial infection has been reported to stimulate hepcidin synthesis^35^. Such stimulation is thought to rely on inflammation-related pathways, although it is not necessarily IL-6 dependent^36^. In our studies, the CHB group had significantly higher HBV-DNA level than the other two patient groups, and their HBV load was positively associated with hepcidin levels, both in the CHB group and in all HBV-infected patients (Fig. 2a). Multiple linear regression analysis also recognized HBV-DNA level as an independent predictor of hepcidin in all the patient groups, especially the CHB group. These data imply that the elevated hepcidin may be ascribed to viral activity. In our cohort, CHB group showed higher average level of the liver inflammation indicator ALT, while the serum CRP level of the CHB group was not significantly different from the control group. However, there are cases in which CRP levels do not correspond with known inflammation-related hepcidin agonists, such as IL-6^37^, so other inflammation-related factors may be responsible for the observed hepcidin upregulation. Given that HBV replication is reported to increase in iron-treated HepG2 cells^38^, and decrease in iron-depleted cells^39^, elevated hepcidin in patients with active CHB may serve to suppress HBV replication through decreasing body iron levels.

Increased tissue and systemic iron levels also stimulate hepcidin production. As an iron regulatory peptide hormone, hepcidin is able to respond to variations in body iron demand and its concentration varies accordingly^32^. Our data showed that both serum iron and ferritin levels were higher in CHB patients than in controls, and that serum hepcidin was positively associated with ferritin in all patients (Fig. 2a). These data indicate that hepcidin synthesis in CHB patients may be responding to elevated iron, in addition to infectious stimuli. This was supported by multiple linear regression which identified ferritin as an independent hepcidin predictor. Higher ferritin levels themselves may be indicative of inflammation, but if inflammation was the only factor operating, serum iron levels would be expected to be reduced.

Available data suggest that HBV and HCV have different effects on hepcidin, with HBV increasing hepcidin expression^22^ and HCV decreasing it^40^,^41^. A possible explanation of this difference is provided by Fujita *et al*.^23^, who suggested that chronic viral hepatitis suppresses hepcidin synthesis by inducing oxidative DNA damage. The oxidative damage caused by HBV is relatively mild compared to that of HCV^23^.

Our results also suggest that patients with HBV-related cirrhosis have a lower mean hepcidin level than those without cirrhosis or those with HBV-related HCC. Several previous studies have also demonstrated that serum hepcidin (or prohepcidin) levels are significantly lower in patients with HBV-related cirrhosis than in those without cirrhosis or healthy subjects^16^,^21^,^24^. One possible reason of this is the reduced liver synthetic capacity that resulted from tissue damage in cirrhosis patients^24^, given that the liver is the primary hepcidin producing organ^32^. Consistent with this hypothesis, the cirrhosis group in our study had the lowest serum albumin concentration, and reduced albumin is widely used as an indicator of liver damage^42^. Albumin was suggested to be an independent predictor of hepcidin in this study by multiple linear regression analysis. Furthermore, hepcidin levels in patients with Child-Pugh-class C HBV-related cirrhosis were lower than patients with Child-Pugh-class B or A, consistent with hepcidin levels reflecting the degree of liver dysfunction. However, the correlations of hepcidin with both viral load and ferritin within this group implies that their hepcidin is still responsive to infectious stimuli and iron, and the decreased viral activity in cirrhosis patients suggested by their generally lower HBV DNA load compared to the CHB group may be seen as an additional factor that down-regulated their serum hepcidin. Although it should be interpreted with caution, an earlier study found that serum prohepcidin correlated inversely with Child-Pugh score^24^. Nonetheless, how hepcidin responds to diverse stimuli may vary according to the insult, as another study suggested that serum hepcidin levels depended on the aetiology of cirrhosis, but not the severity^43^.

Data on hepcidin synthesis in HBV-related HCC are even more limited than they are for CHB and cirrhosis. Several studies have reported that hepcidin mRNA expression is lower in liver carcinoma tissue than in adjacent non-tumor tissue of HCC patients^44^,^45^,^46^, but one of these also found that hepcidin mRNA was overexpressed in the non-tumor tissue of HBV-HCC patients compared to normal liver controls^44^. This was attributed to hepatic iron accumulation. Why serum hepcidin is upregulated in the HBV-HCC cohort is unclear. There is a close correlation between inflammation and HCC^47^, and in our study CRP levels were higher in the HCC group than in the cirrhosis group, although the difference was not statistically significant. Furthermore, the HCC group also showed significantly higher serum iron and ferritin than control group, and iron is necessary for the proliferation of neoplastic cells^48^. Hence inflammation and iron loading may both have a role in the elevation of hepcidin in HBV-HCC patients. Since iron overload itself is associated with HCC risk, the reduction of hepcidin during the cirrhosis stage may play a role in facilitating HCC formation. Further exploration on hepcidin production in patients with HCC would be needed to elaborate this issue.

In conclusion, this work has successfully applied a recently established nanopore silica film based hepcidin assay on serum samples of HBV-infected patients at different disease stages. We found that serum hepcidin levels in patients with CHB and HBV-related HCC are significantly higher than in healthy controls and patients with HBV-related cirrhosis. Serum hepcidin positively correlated with HBV-DNA load and serum ferritin in all the patients, indicating that viral infection and iron loading were important simulators of hepcidin synthesis in HBV-related diseases. In cirrhotic patients, hepcidin synthesis may be decreased by the impaired synthetic capacity of liver. This is the first study to investigate the variations of serum hepcidin in all three major stages of chronic HBV infection, and to demonstrate the possible association between hepcidin and HBV-related disease progression. We believe that this work further confirmed the utility of our nanopore film assay in clinical research, and that by adding to the current understanding of iron disorders in HBV infections, these observations may provide new insights into development of new approaches for HBV infection surveillance. However, hepcidin regulation and its disruption by HBV infection is highly complicated, and further studies with larger cohorts are required to explore the issue.

## Methods

### Materials

All chemicals and reagents are purchased from Sigma-Alderich (St. Louis, MO, USA) unless specified otherwise. Synthetic human hepcidin was obtain from Peptides Institute (Osaka, Japan). MALDI-TOF MS matrix α-cyano-4-hydroxycinnamic acid was from Bruker Daltonics (Billerica, MA, USA). Nanopore silica film coated wafer chips for serum hepcidin enrichment were fabricated as described previously^14^.

### Patients

Forty-six HBV-infected patients admitted to the First Affiliated Hospital of Jilin University from 2010 to 2011 were studied (Table 1). Of these, 16 had active infection but no sign of cirrhosis (by computerized tomography) and no history of anti-viral treatment, 14 had HBV-related cirrhosis, and 16 had HBV-HCC. HBV infection was diagnosed through detection of HBV surface antigen and viral DNA. All patients were anti-hepatitis C virus (HCV) negative. Cirrhosis and HCC were diagnosed through computerized tomography or magnetic resonance imaging. In the HBV-related cirrhosis group, the degree of liver injury was recorded according to the Child-Pugh classification (5 Child A, 5 Child B and 4 Child C). Patients with other potential causes of hepcidin dysregulation, including anemia, hemochromatosis, autoimmune hepatitis, primary biliary cirrhosis, renal failure or other kinds of metabolic syndrome were excluded. The control group included 20 age- and sex-matched healthy volunteers, all of whom had normal aminotransferase and iron levels, and all were HBV surface antigen-negative. All studies were carried out in accordance with the Declaration of Helsinki and other relevant guidelines and regulations, and were approved by the Human Ethics Committee of the First Affiliated Hospital of Jilin University. Informed consent was obtained from all participants.

### Blood collection and hepcidin measurements

Blood was collected from 6:00–7:30 a.m. after overnight fasting. Whole blood was used for hemoglobin determination. For other parameters, blood was allowed to clot, then centrifuged at 2300 g for 10 minutes. The supernatants were transferred to new tubes, aliquoted and stored at −80 °C until use. For hepcidin measurements, serum samples were processed by the nanopore silica film coated chips developed by our laboratory and quantified by MALDI-TOF MS using the peptide ACTH 18–39 as internal standard. A detailed procedure of sample processing, method calibration and hepcidin quantification is described in our preceding publication^14^.

### Laboratory methods for blood indices

Blood biochemical indices, including liver enzymes (alanine aminotransferase, aspartate aminotransferase, alkaline phosphatase), serum total bilirubin, albumin, hemoglobin, and C-reactive protein (CRP) were determined by standard laboratory methods. HBV surface antigen was detected by a commercially available immunoassay. HBV-DNA was detected by polymerase chain reaction. Serum ferritin was measured by radioimmunoassay. Serum iron and total iron-binding capacity were measured by colorimetry. All clinical laboratory assays were carried out by the Clinical Laboratory of the First Affiliated Hospital of Jilin University.

### Statistical analysis

Data were analyzed using SPSS 13.0. The Kolmogorov-Smirnov test was applied to check the normal distribution of the quantitative variables. Data are presented as mean ± standard deviation (SD) or medians (with interquartile range). Categorical variables were compared using Pearson’s χ^2^ test. According to variable distribution, one-way ANOVA or non-parametric Kruskal-Wallis test was used for multi-group comparisons. Correlation coefficients (r) were evaluated between hepcidin and all the variables using Pearson’s or non-parametric Spearman’s correlation analysis according to variable distribution. Multiple linear regression analysis was employed to evaluate whether other potential confounders affected the association of hepcidin and ferritin. A *p* value of <0.05 was considered statistically significant.

## Additional Information

**How to cite this article**: Wang, J. *et al*. Correlation of serum hepcidin levels with disease progression in hepatitis B virus-related disease assessed by nanopore film based assay. *Sci. Rep*. **6**, 34252; doi: 10.1038/srep34252 (2016).

## Figures and Tables

**Table 1:** Demographics and clinical profiles of HBV-infected patients and healthy controls.

Parameters	Chronic hepatitis B (N = 16)	HBV-related cirrhosis (N = 14)	HBV-related cirrhosis with HCC (N = 16)	Control (N = 20)
Age (years)	41 ± 12.0	49.6 ± 14.8	54.9 ± 6.8	49 ± 9.0
Gender (M,F)	13, 3	10, 4	14, 2	16, 4
AST (U/L) ^*^	134.7 ± 114	165.3 ± 243.5	90.5 ± 52.3	8–40
ALT (U/L) ^*^	309.4 ± 311.3^d^	195.6 ± 397.1	75.3 ± 54.4	8–50
ALP (U/L) ^*^	112.3 ± 34.4	121.3 ± 57.0	168.8 ± 98.4	15–112
TBIL (μmol/L) ^*^	23.3 (9.3–613.5)	25.1 (11.1–410.4)	33.6 (12.9–593.9)	6.8–30
Albumin (g/L) ^*^	37.3 ± 4.9^c,d^	28.3 ± 4.7^b^	32.3 ± 4.8^b^	35–55
Hemoglobin (g/L)	147.5 ± 12.5^c^	125.7 ± 17.5^b^	137.3 ± 22.2	138 ± 15
HBV-DNA (10^4^ IU/mL) ^*^	244 (4.3–32100)^c,d^	1.9 (1.05–8720)	26.1 (0.38–346)	0–0.05
Serum iron (μmol/L)	29.4 ± 7.3^a^	22.1 ± 13.6^a^	27.6 ± 6.9^a^	18.6 ± 4.4
TIBC (μmol/L)	60.8 ± 10.7	55.7 ± 11.6	54.3 ± 12.6	75.8 ± 13.7
Ferritin (ng/mL)	672.7 ± 675.2^a,c^	320.6 ± 237.1^a,b,d^	501.5 ± 583.3^a,c^	190 ± 163
CRP ^*^	4.6 ± 5.0	4.4 ± 4.6	4.9 ± 6.3	0–6

Data are summarized as mean ± SD or, in some cases, as median (interquartile range). According to the normality of variable distribution tested by the Kolmogorov-Smirnov test, one-way ANOVA or non-parametric Kruskal-Wallis test was performed for *p* values. ^a^*p* < 0.05 vs. control; ^b^*p* < 0.05 vs. chronic hepatitis B group; ^c^*p* < 0.05 vs. cirrhosis group; ^d^*p* < 0.05 vs HCC group. *Reference range given as control values. Abbreviations: HBV, hepatitis B virus; HCC, hepatocellular carcinoma; ALT, alanine aminotransferase; AST, aspartate aminotransferase; ALP, alkaline phosphatase; TBIL, total bilirubin; TIBC, iron and total iron-binding capacity; CRP, C-reactive protein.

**Table 2:** Correlation between hepcidin and all variables in the HBV-related patients.

Parameters	Chronic hepatitis B	HBV-related cirrhosis	HBV-related cirrhosis with HCC	Total patients	*r*	*p*-value	*r*	*p*-value
	*r*	*p*-value	*r*	*p*-value				
AST (U/L)	0.03	—	−0.19	—	0.11	—	0.05	—
ALT (U/L)	0.07	—	0.44	—	0.12	—	0.05	—
ALP (U/L)	−0.13	—	−0.2	—	0.08	—	0.06	—
TBIL (μmol/L)	−0.18	—	−0.17	—	−0.09	—	0.07	—
Albumin (g/L)	0.08	—	−0.49	—	0.33	—	0.32	<0.05
Hemoglobin (g/L)	0.16	—	−0.10	—	0.11	—	0.26	—
HBV-DNA (IU/mL)	0.76	<0.01	0.60	<0.05	0.55	—	0.48	<0.01
Serum iron (μmol/L)	0.33	—	0.40	—	−0.06	—	0.13	—
TIBC (μmol/L)	−0.06	—	−0.51	—	0.21	—	−0.11	—
Ferritin (ng/mL)	0.41	—	0.56	<0.05	0.79	<0.01	0.62	<0.001
CRP (ng/mL)	−0.28	—	−0.46	—	0.14	—	−0.06	—

Pearson’s or Spearman’s correlation analysis was performed for *r* and *p* values according to variable distribution. Abbreviations: HBV, hepatitis B virus; HCC, hepatocellular carcinoma; ALT, alanine aminotransferase; AST, aspartate aminotransferase; ALP, alkaline phosphatase; TBIL, total bilirubin; TIBC, iron and total iron-binding capacity; CRP, C-reactive protein.

**Table 3:** Results of multiple linear regression models for serum hepcidin concentration.

Parameters	β	HBV-related patients		*p*-value
		95%CI		
		Lower limit	Upper limit	
Age (years)	0.237	—	—	—
AST (U/L)	−0.152	—	—	—
ALT (U/L)	−0.231	—	—	-
ALP (U/L)	−0.367	—	—	—
TBIL (μmol/L)	−0.137	—	—	—
Albumin (g/L)	0.363	0.065	0.565	0.017
Hemoglobin (g/L)	−0.293	—	—	—
HBV-DNA (IU/mL)	0.702	0.000	0.001	0.005
Serum iron (μmol/L)	−0.129	—	—	—
TIBC (μmol/L)	−0.244	—	—	—
Ferritin (ng/mL)	0.516	0.003	0.011	0.002

Multiple linear regression was performed for β and *p* values. Abbreviations: HBV, hepatitis B virus; HCC, hepatocellular carcinoma; ALT, alanine aminotransferase; AST, aspartate aminotransferase; ALP, alkaline phosphatase; TBIL, total bilirubin; TIBC, iron and total iron-binding capacity.
